# Zika virus dysregulates human Sertoli cell proteins involved in spermatogenesis with little effect on tight junctions

**DOI:** 10.1371/journal.pntd.0008335

**Published:** 2020-06-08

**Authors:** Mahamud-ur Rashid, Ali Zahedi-Amiri, Kathleen K. M. Glover, Ang Gao, Michaela E. Nickol, Jason Kindrachuk, John A. Wilkins, Kevin M. Coombs

**Affiliations:** 1 University of Manitoba, Department of Medical Microbiology and Infectious Diseases, Winnipeg, Manitoba, Canada; 2 Manitoba Centre for Proteomics & Systems Biology, Winnipeg, Manitoba, Canada; 3 University of Manitoba, Department of Internal Medicine, Health Sciences Centre, Winnipeg, Manitoba, Canada; 4 Children’s Hospital Research Institute of Manitoba, John Buhler Research Centre, Winnipeg, Manitoba, Canada; Molecular Biology Unit (MBU), INDIA

## Abstract

Zika virus (ZIKV), a neglected tropical disease until its re-emergence in 2007, causes microcephaly in infants and Guillain-Barré syndrome in adults. Its re-emergence and spread to more than 80 countries led the World Health Organization in 2016 to declare a Public Health Emergency. ZIKV is mainly transmitted by mosquitos, but can persist in infected human male semen for prolonged periods and may be sexually transmitted. Testicular Sertoli cells support ZIKV replication and may be a reservoir for persistent ZIKV infection. Electrical impedance analyses indicated ZIKV infection rapidly disrupted Vero cell monolayers but had little effect upon human Sertoli cells (HSerC). We determined ZIKV-induced proteomic changes in HSerC using an aptamer-based multiplexed technique (SOMAscan) targeting >1300 human proteins. ZIKV infection caused differential expression of 299 proteins during three different time points, including 5 days after infection. Dysregulated proteins are involved in different bio-functions, including cell death and survival, cell cycle, maintenance of cellular function, cell signaling, cellular assembly, morphology, movement, molecular transport, and immune response. Many signaling pathways important for maintenance of HSerC function and spermatogenesis were highly dysregulated. These included IL-6, IGF1, EGF, NF-κB, PPAR, ERK/MAPK, and growth hormone signaling. Down-regulation of the PPAR signaling pathway might impact cellular energy supplies. Upstream molecule analysis also indicated microRNAs involved in germ cell development were downregulated by infection. Overall, this study leads to a better understanding of Sertoli cellular mechanisms used by ZIKV during persistent infection and possible ZIKV impacts on spermatogenesis.

## Author summary

Zika virus has gained new significance during the past few years when it became associated with increased numbers of birth defects. Zika virus is primarily transmitted by mosquitoes. However, it is becoming increasingly clear that there are other ways the virus can be transmitted. One of these ways is through sexual transmission. In order to better understand how Zika virus can persist in humans and may be transmitted sexually, we examined the interactions between Zika virus and Sertoli cells, a specialized male testicular cell responsible for sperm production. We examined how Zika virus infection affected more than 1300 Sertoli cell proteins and found that virus infection altered cellular pathways responsible for sperm production. At the same time, Zika virus had little effect upon overall structure of the cells. This information helps better explain the long-lived persistence of Zika virus in human male reproductive cells and the virus’ capacity to be sexually transmitted.

## Introduction

Zika virus (ZIKV) is considered a neglected tropical disease. It is a small enveloped arthropod-borne human pathogen (arbovirus) with a small positive-sense single-stranded RNA genome. ZIKV belongs to the family *Flaviviridae*, which includes other human pathogens such as West Nile virus (WNV), Dengue virus (DENV), Yellow fever virus (YFV), and Japanese encephalitis virus (JEV) [1]. ZIKV was discovered in 1947 in a sentinel monkey [2], and first isolated from humans in 1952 [3].

Followed by a few sporadic infections in Pakistan and Malaysia reported in 1977, ZIKV re-emerged in the Pacific islands in 2007 and spread to more than 80 countries/territories worldwide including regions in Latin America, USA, and Southeast Asia [4–6]. In February 2016, the World Health Organization (WHO) declared ZIKV a Global Health Emergency [7]. Because of the non-specific nature of ZIKV disease symptoms and that >80% of patients remain asymptomatic, the virus was neglected for a long time [8]. The virus received recent major attention due to its association with Guillain-Barré syndrome in adults and microcephaly in newborns [3, 4, 8].

ZIKV is mostly transmitted by *Aedes* mosquitoes (*A*. *aegypti* and *A*. *albopictus*) in endemic areas [9], but sexual transmission was suggested from traveler-associated infections in non-endemic countries/territories [7, 10–12]. ZIKV can persist in infected male semen for prolonged periods in the absence of viremia and disease symptoms. Live ZIKV and viral RNA were detected in semen up to 370 days after disease onset [13–15]. ZIKV infection causes severe pathological effects in male murine testicular tissues and reduces sperm motility and fertility [16, 17]. *In vitro* studies demonstrated that ZIKV can infect human testicular tissue and replicate in human Sertoli cells (HSerC), which may be a reservoir for long-term viral persistence [16, 18, 19].

Sertoli cells are considered the “mother” for spermatogonial stem cells, as they play critical roles in testis formation and aid the spermatogenesis process by supplying nutrients and developmental signals [20]. They also provide structural support to germ cells and create the blood-testis barrier (BTB) to ensure an immune-privileged environment to protect sperm cells from immune attack [21]. Unlike other bodily cells, HSerC depend upon fatty acid (FA) oxidation rather than glucose for energy [22]. The Peroxisome Proliferator-Activated Receptor (PPAR) signaling pathway plays an important role in energy generation by lactate and lipid oxidization [23]. Sertoli cells can influence the regulation of hormones, growth factors, and receptor signaling important for spermatogenesis and human fertility [24]. Follicle-stimulating hormone (FSH) is one of the key endocrine hormones that directly regulates spermatogenesis [25]. Sertoli cells secrete inhibin B after induction by FSH [26], but the level of inhibin B also provides negative feedback on FSH secretion [27]. The level of serum inhibin B directly correlates with sperm count and is used as a marker for spermatogenesis [27].

*In vitro* and *in vivo* studies demonstrated that ZIKV persists in Sertoli cells [16, 18], but the mechanism(s) underlining persistence is/are unknown. Although Sertoli cells play many critical roles in germ cell development, the impact of ZIKV infection on the spermatogenesis process is not well understood. In this study, we determined the impact of ZIKV infection on proteins known to be important for HSerC function and spermatogenesis.

We used Slow Off-rate Modified Aptamers (SOMAmers; SomaLogics, Inc., Denver, CO), a multiplexed proteomic technique that measures 1305 unique proteins in up to 88 different samples simultaneously with high precision [28], and found that numerous proteins involved in multiple bio-functions were dysregulated. Bioinformatic analyses predict that many of these proteins, and upstream regulatory elements, are involved in germ cell development and spermatogenesis, which has important implications for male fertility and reproduction.

## Results

### ZIKV infectivity and cytopathic impact on primary HSerC

**Fig 1:**
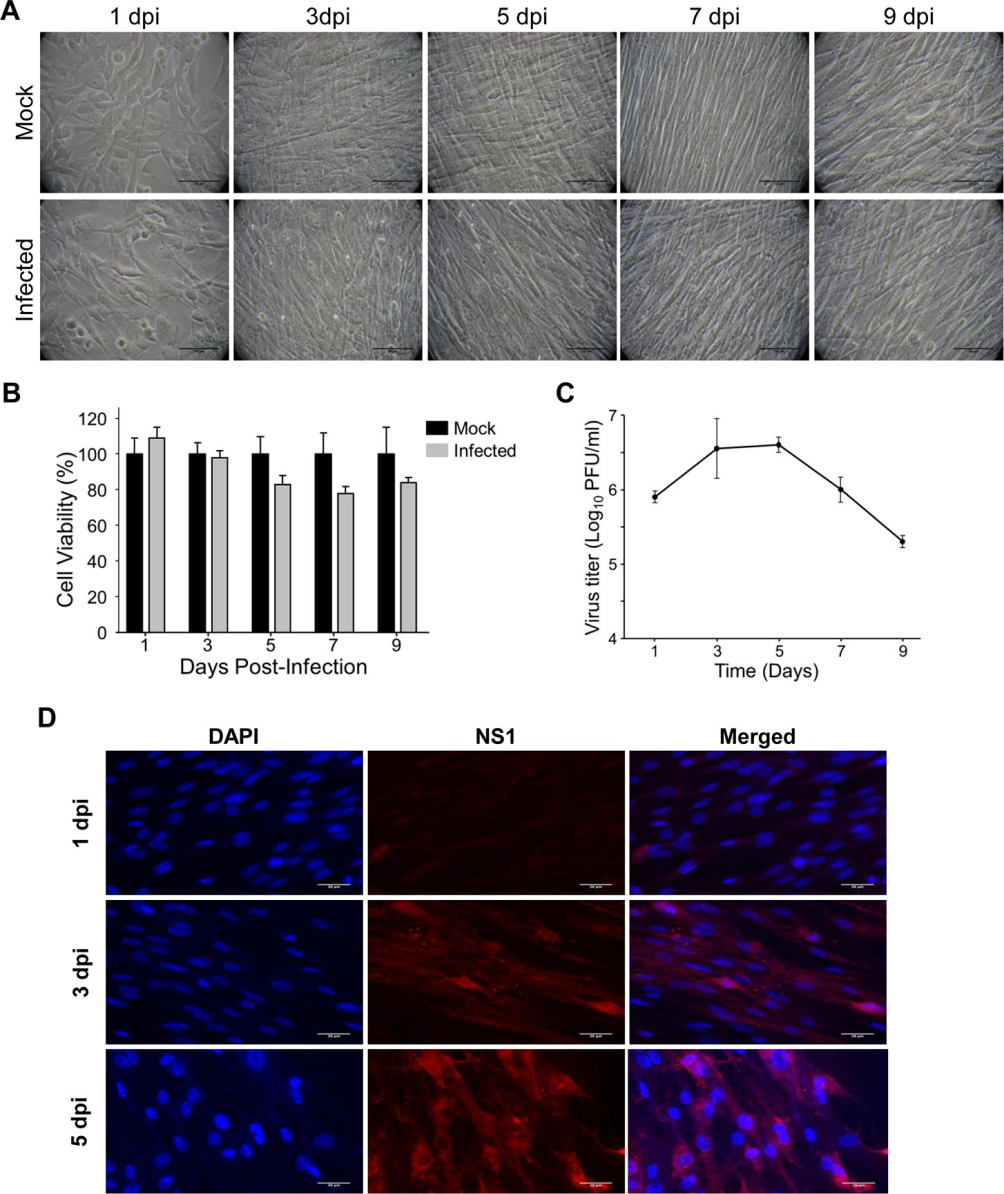
Zika virus (ZIKV) infectivity and cytopathic impact on human primary Sertoli cells (HSerC). HSerC were infected with ZIKV at an MOI of 3. Cell morphology, cell viability, and virus titer were measured every alternate day from day 1 to 9. (A) Cells were visualized under bright-field microscopy at 200× magnification and assessed for cytopathic effect. Scale bar is 50 μm. (B) HSerC viability was measured after ZIKV infection by WST-1 assay. (C) Virus titer in the infected cell supernatant was determined by plaque assay. The experiment was done in three replicates. (D) HSerCs were fixed after Day 1, 3 and 5 days post ZIKV infection, and viral protein expression was determined by fluorescent microscopy using ZIKV-NS1 monoclonal antibodies. Images were taken at 200× magnification. Scale bar is 20 μm. Cell nuclei were visualized by DAPI stain. dpi = days post-infection.

Primary HSerC were infected with ZIKV at MOI = 3, then virus infectivity and impact on cells were monitored up to 9 days post-infection (dpi). There was no observable ZIKV-induced cytopathic effect (CPE) throughout the tested infection course (Fig 1A). Cell viabilities declined to approximately 80% on days 5, 7 and 9 but were not statistically different from non-infected mocks (Fig 1B). Supernatant virus titers were determined by plaque assay. The number of progeny viruses started rising by 1 dpi, reached a peak of 4.4 × 10^6^ PFU/ml by 5 dpi, but dropped below 10^5^ PFU/ml by 7 and 9 dpi (Fig 1C). The rise in viral titer also corresponded with increased appearance of viral non-structural protein 1 (NS-1; Fig 1D), a marker of productive infection.

**Fig 2:**
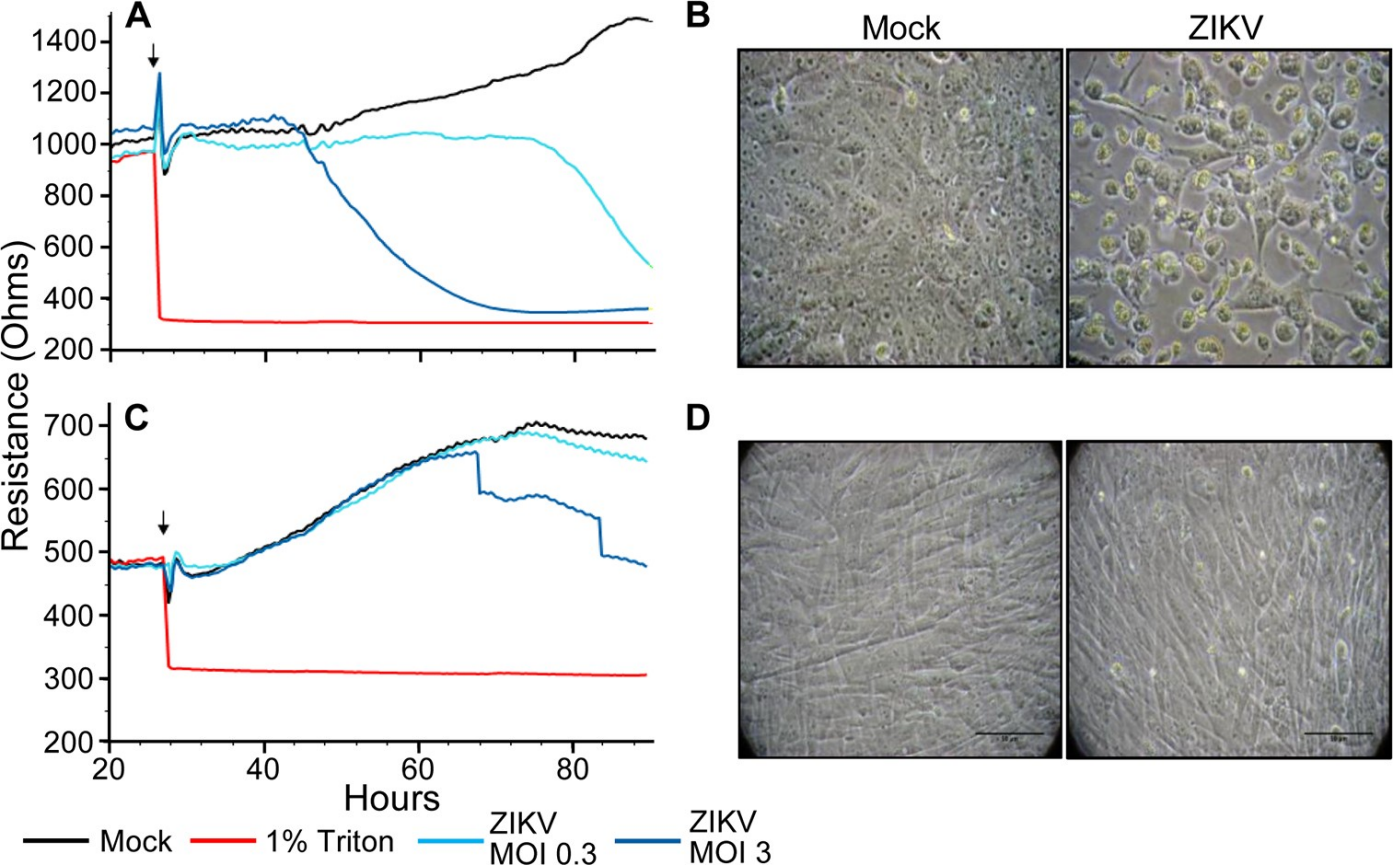
**Electrical impedance of ZIKV-infected Vero (A) and HSerC (C)**. Cells were added to 96-well ECIS plates and allowed to grow for 24h to create monolayers. Cells were then treated, at time indicated with small vertical arrows, with media alone (positive control; black line), 1% Triton X-100 (negative control; red), or with ZIKV at MOI of 0.3 (teal) or 3.0 (dark blue) and cultures maintained with continuous impedance monitoring for another 66–80 hours. Values represent the averages obtained from 8 wells for each condition, and HSerC were analyzed twice. Micrographs of Vero (**B**) and Sertoli (**D**) cells at 72 hpi demonstrating observable CPE.

The lack of apparent ZIKV-induced HSerC CPE even after 9 days, and the function of HSerC to form the BTB led us to more quantitatively assess the impact of virus infection on cellular barrier function. We used electric cell-substrate impedance sensing (ECIS) to obtain real-time measurements of impedance and of CPE. We also used Vero cells, which, although they do not normally form tight-junction barriers, are highly susceptible to ZIKV and experience rapid CPE. The Vero cells experienced a total loss of electrical resistance by 75 hpi when infected at MOI 0.3, and lost resistance sooner at a higher MOI of 3 (Fig 2A). Disruption was also observed microscopically (Fig 2C). Conversely, HSerC infection with ZIKV at MOI 0.3 resulted in no changes in resistance during the first 47 h of infection, and only a small loss in resistance at 51 hpi (75h post-plating), which mimicked the trend seen in mock-infected cells (Fig 2B). HSerC infected with ZIKV at higher MOI of 3.0 showed no loss in resistance until 40 hpi (64h post-plating) and only moderate loss of resistance later, confirming that ZIKV infection of HSerC has only moderate effects on cell barrier integrity, which also was reflected by considerably lower CPE (Fig 2D). Based on these collective observations, we selected day 1 (early), day 3 (mid) and day 5 (peak virus titer) for subsequent global proteomic screens.

### Proteomic dysregulation in HSerC after ZIKV infection

**Fig 3:**
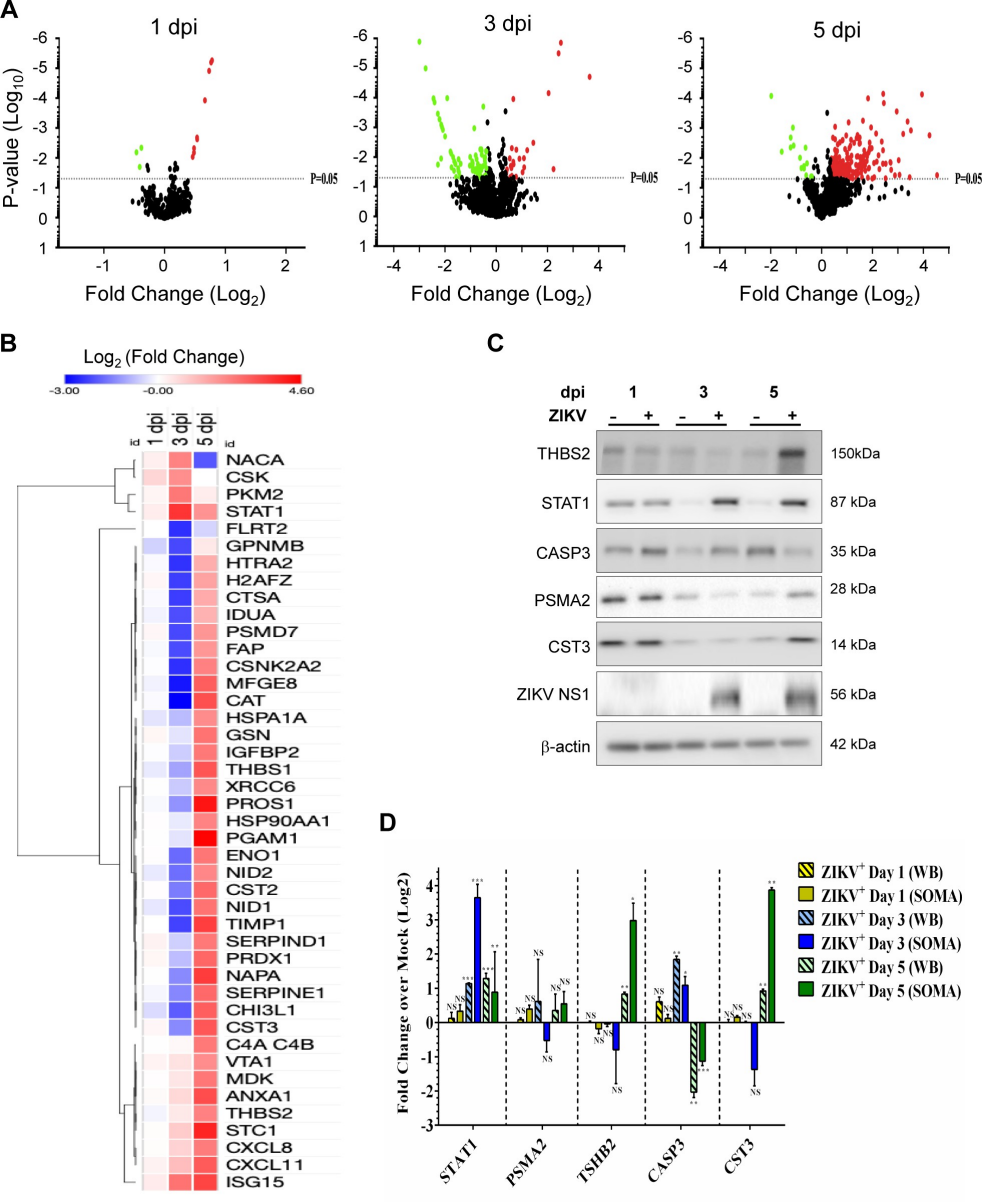
Proteomic analyses of ZIKV-infected HSerC by SOMAscan. (**A**) Volcano plots showing the expression fold changes and the significance of differentially expressed proteins at 1, 3 and 5 dpi (P-value < 0.05). Up- and down-regulated proteins are highlighted in red and green, respectively. (**B**) Heat map of selected differentially expressed (fold change > 4 or < -4) proteins at 1, 3 and 5 days post ZIKV infection. Up- and down-regulated proteins are highlighted in red and blue, respectively. (**C**) Validation of SOMAscan results by Western blot of 5 selected differentially regulated proteins. (**D**) Expression value of the proteins from Western-blot were quantified from three replicates and plotted side-by-side to SOMAscan expression values. In both techniques, expression value of each protein in infected cells was compared with time-matched mock for quantifying significant dysregulation. *** = P-value <0.001, ** = P-value < 0.01, * = P-value < 0.05. dpi = days post-infection.

We used SOMAscan, a multi-plexed targeted system that can detect 1305 proteins simultaneously from each of up to 88 samples, to examine ZIKV infection-induced HSerC proteomic dysregulation. Samples were collected at 1, 3, and 5 dpi and time-matched non-infected mock samples, from each of three biological replicates. P values were determined by T-test and Z-scores, as detailed in Materials and Methods. A total of 358 proteins were deemed significantly dysregulated at any time point (Table 1). Almost 300 proteins were significantly dysregulated more than 1.3-fold, and more than 125 proteins were significantly dysregulated more than 2-fold. Irrespective of whichever fold-change cut-offs we choose, we generally found that most dysregulated proteins were downregulated at 3 dpi, whereas most were upregulated at 5 dpi (Table 1; Fig 3A and 3B). For subsequent bioinformatics analyses we considered fold-change cut-offs of ± 1.3-fold and fold-change cut-offs of ± 1.5-fold, both with p < 0.05, compared to mock. Apart from differences in numbers of significantly considered proteins at these two cut-off levels, there were no major changes in identified pathways, networks and bio-functions; thus, for more completeness, we consider significant cut-offs of ± 1.3-fold below. Collectively, 299 proteins were significantly dysregulated at any of the three different time points, with 12 (9 up- and 3 down-regulated) dysregulated at 1 dpi, 95 (25 up- and 70 down-regulated) dysregulated at 3 dpi, and 218 (201 up- and 17 down-regulated) dysregulated at 5 dpi (Table 1, Fig 3A, S1 Table). Protein abundance heat maps and clustering analysis showed there were 14 significantly down-regulated proteins at day 3 dpi that were significantly up-regulated at 5 dpi, whereas 3 proteins were up-regulated at both time points (Fig 3B; Table 1; S1 Fig). We validated several proteins’ dysregulation by Western blot, based on high fold-changes and antibody availability (Fig 3C) and confirmed similar patterns of expression (Fig 3D). For example, STAT1 was not significantly dysregulated by 1 dpi as measured by both SOMAscan and immunoblot, and, although absolute fold-change values differed between SOMAscan and immunoblot, both methods indicated STAT1 was significantly up-regulated by 3 and 5 dpi.

**Table 1:** Numbers of significantly dysregulated ZIKV-infected HSerC proteins.

Number that are significant	Total Unique	1 dpi	3 dpi	5 dpi
and Fold-change > 1.00	358	16	46	213
and Fold-change < -1.00		5	90	21
and Fold-change > 1.10	354	13	45	212
and Fold-change < -1.10		5	89	21
and Fold-change > 1.30	299	9	25	197
and Fold-change < -1.30		5	67	17
and Fold-change > 1.50	216	4	19	152
and Fold-change < -1.50		0	51	12
and Fold-change > 2.00	128	0	10	100
and Fold-change < -2.00		0	30	6
and Fold-change > 3.00	71	0	5	53
and Fold-change < -3.00		0	21	1

Significance was determined by T-test and Z-score (p < 0.05) as detailed in Methods

### ZIKV dysregulates cellular functions and signaling pathways in HSerC

The list of significantly dysregulated proteins was uploaded into IPA to determine ZIKV-induced impacts on cellular protein networks, signaling pathways and cellular functions. IPA could not predict any networks with confidence at 1 dpi because of the low numbers of dysregulated molecules. A total of 11 networks were predicted by IPA at 3 dpi (n = 5) and at 5 dpi (n = 6) that had scores >20 and comprised 11 or more focus molecules (S2 Table, S2 Fig). Changes in protein expression as a function of progressing infection were constructed for three of these networks (Cell-to-cell signaling, Cell death and survival, and Post-translational modification; S3 Fig) and demonstrate little, if any dysregulation at 1 dpi, general down-regulation at 3 dpi and general activation at 5 dpi. Proteins expressed differentially after ZIKV infection at 3 dpi and 5 dpi were classified into three functional categories, including biological processes, cellular components, and molecular functions by GOTERM and PANTHER. A wide range of biological process subcategories were enriched at 3 dpi, with some of the highest enrichment scores from up-regulated proteins attributed to JAK-STAT cascade, chromatin assembly, and negative regulation of apoptotic process (S4 Fig). The vast majority of these biological processes were not enriched later at 5 dpi; instead up-regulated proteins at this later time showed higher enrichment for entirely different classes of biological processes, such as gluconeogenesis, angiogenesis, glycolysis, and protein phosphorylation. Similar differential biological process enrichment patterns also were observed for down-regulated proteins, and in molecular functions at 3 dpi compared to 5 dpi. Cellular growth and proliferation, post-translational modification, cellular development, and cell signaling were significantly decreased and immune cell trafficking, and cell death by apoptosis were significantly increased at 3 dpi (S3 Table).

**Fig 4:**
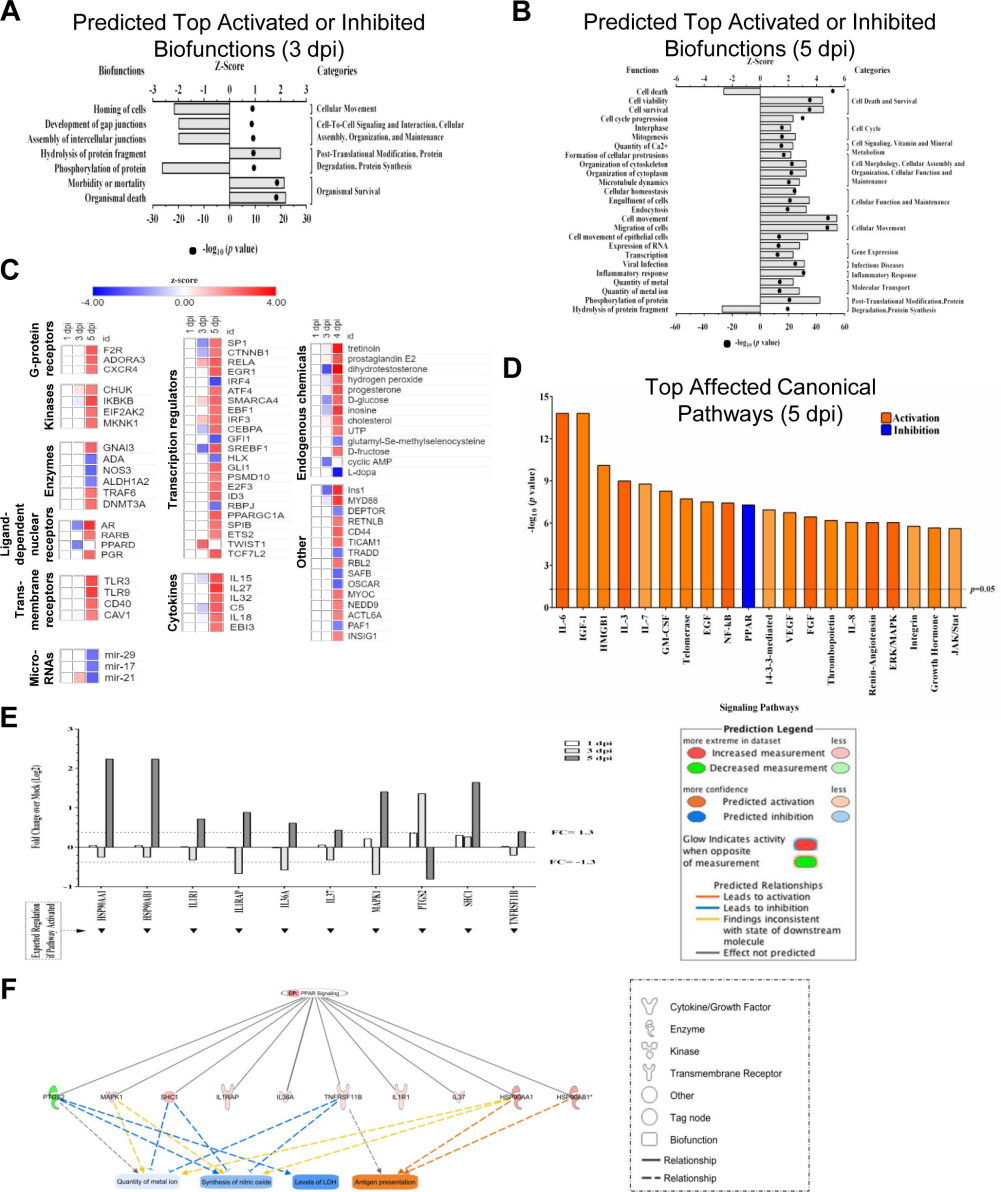
IPA-predicted activation and inhibition of bio-functions, upstream molecules, and canonical pathways. **(**A) Top bio-functions and predicted activation or inhibition Z-Scores (upper x-axis) and Log_10_ p-values (lower x-axis) are indicated at 3 dpi and (B) 5 dpi. Activation is indicated by positive Z-Score and the inhibited bio-functions are indicated by negative Z-scores. Major bio-function categories are indicated at right. (C) Top upstream molecules and prediction of their activation or inhibition based on Z-Scores. (D) Top affected canonical pathways with Log_10_ p-value indicated at the top. Orange corresponds to activation, and blue corresponds to inhibition, with the degree of coloration corresponding to Z-Score. (E) ZIKV-induced inhibition of selected proteins within the PPAR signaling pathway as a function of time post-infection. Note that by 5 dpi, all proteins are dysregulated ≥ 1.3-fold. (F) Predicted effects of indicated PPAR pathway proteins on various bio-functions.

Bio-function analysis by IPA also predicted that development of gap junctions, assembly of intercellular junctions and protein phosphorylation were significantly downregulated, whereas protein fragment hydrolysis and organismal death were increased (Fig 4A). At 5 dpi, cell death by necrosis, post-translational modification and protein synthesis were significantly decreased and cellular movement, immune cell trafficking, cell viability, cell-to-cell signaling, cell proliferation, cell cycle, cellular development and maintenance, cellular assembly and inflammatory response were significantly increased (Fig 4B, S4 Table).

Based on the dysregulated proteins, IPA predicted a total of 383 canonical pathways that were significantly affected by 5 dpi. Among them, the top 20 pathways are Il-6, IGF-1, HMGB1, IL-3, IL7, GM-CSF, Telomerase, EGF, NF-kB, PPAR, 14-3-3 mediated, VEGF, FGF, Thrombopoietin, IL-8, Renin-Angiotensin, ERK/MAPK, Integrin, Growth hormone, and JAK/Stat signaling pathways (Fig 4D). The PPAR pathway was the only significantly down-regulated pathway at this time point. HSP90AA1, HSP90AB1, IL1R1, ILRAP, IL36A, IL37, MAPK1, PTGS2, SHC1, and TNFRSF11B, all involved in PPAR pathway, were significantly up-regulated at 5dpi (Fig 4E). Direct and indirect IPA-predicted protein interactions suggest metal ion, synthesis of nitric oxide, and LDH levels will significantly decrease, but antigen presentation will increase in HSerC by 5dpi (Fig 4F). IPA predicted another 205 proteins that were up-regulated and 75 down-regulated by upstream analysis by 5 dpi. These proteins belong to G-protein receptor, kinases, enzymes, transmembrane receptors, transcription regulators, cytokines, microRNAs, and endogenous chemicals. Many of the predicted upstream molecules are involved in maintenance of HSerC function and spermatogenesis (Fig 4C).

Because of the large numbers of dysregulated proteins and pathways described earlier, we then focused on those proteins significantly dysregulated >5-fold. Of the 26 proteins that exceeded this threshold, 20 were dysregulated at 5dpi, 7 at 3 dpi, only 1 (ISG15) was dysregulated >5-fold at both time points, and no proteins were dysregulated >5-fold at 1dpi. The dysregulated proteins belong to cytokines (TIMP1, CXCL11, CXCL8), enzymes (ANXA1, CHI3L1, CHI3L1, CAT), growth factor (MDK), kinases (STC1, PKM), peptidase (HTRA2), phosphatase (PGAM1), transcription regulator (STAT1), transporter (NAPA), and others (S5 Fig). The five top-most dysregulated proteins are phosphoglycerate mutase 1 (PGAM1), protein S (PROS1), stanniocalcin 1(STC1), NSF attachment protein alpha (NAPA) and TIMP metallopeptidase inhibitor 1(TIMP1), with expression levels >11-fold compared to non-infected at 5dpi. Sixteen of the 26 top dysregulated proteins are predicted to affect cell-to-cell signaling and interaction, immunological disease, organismal injury and abnormalities in cellular networks.

### ZIKV disrupts the expression of Inhibin B by HSerC

**Fig 5:**
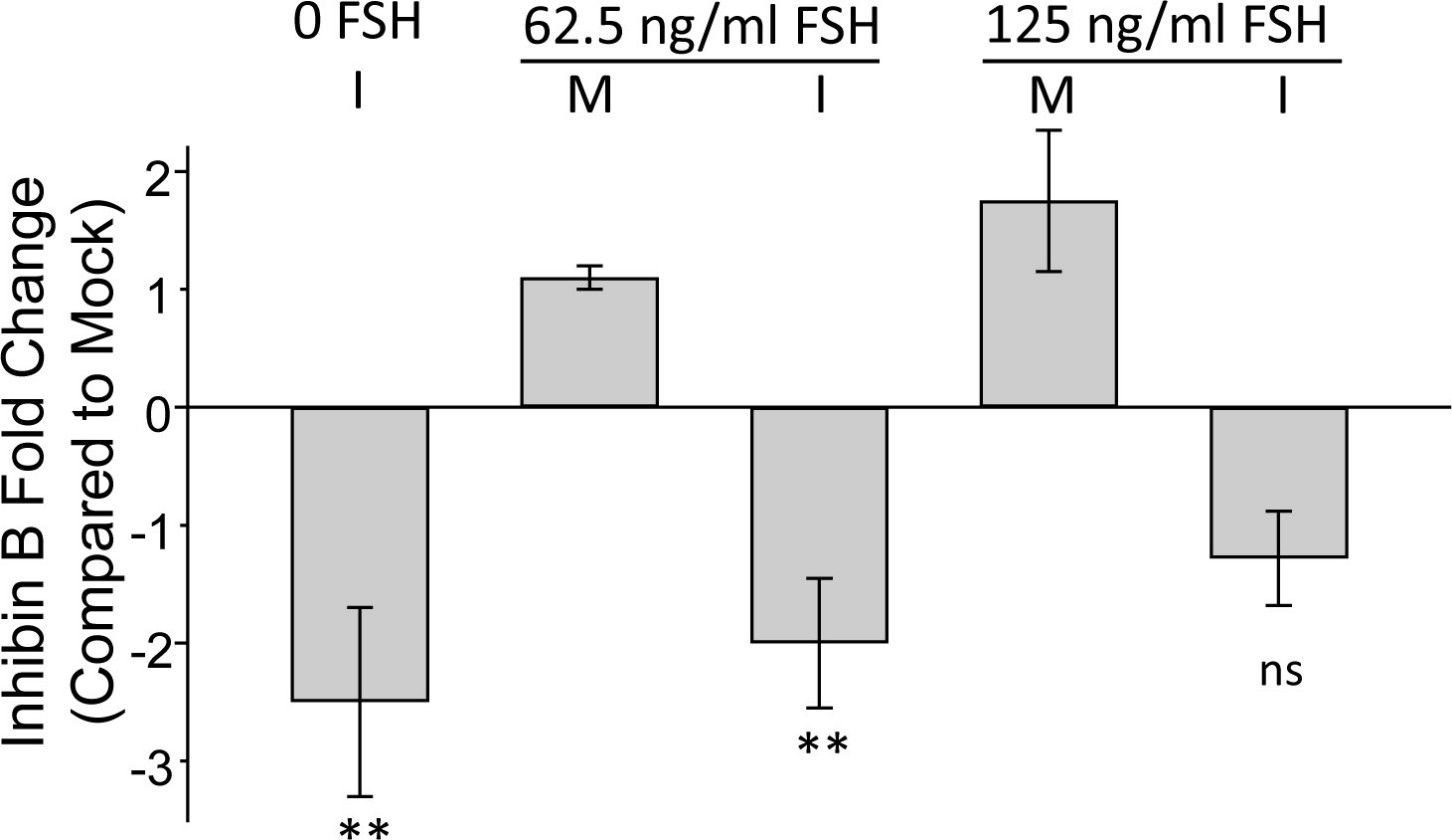
Inhibin B secretion by HSerC after FSH treatment and ZIKV infection. Cells were infected (I) with ZIKV at MOI = 3, or non-infected mock (M), and treated with indicated concentrations of follicular stimulating hormone (FSH) for 48 hours. Impact of infection and FSH on inhibin B production monitored by ELISA and compared to non-infected/non-treated controls. n = 3. ** = p-value < 0.01, * = p-value < 0.05.

In light of the many dysregulated proteins being involved in maintenance of Sertoli cell function and spermatogenesis (Fig 4C) and to better delineate potential functional consequences of ZIKV infection on spermatogenesis, we determined inhibin B levels in culture supernatants after infection and follicular stimulating hormone (FSH) treatment. FSH concentrations of 62.5 and 125ng/ml caused 1.1- and 2.4-fold increases in inhibin B expression, respectively (Fig 5). However, ZIKV infection caused significant reductions in inhibin B expression.

## Discussion

ZIKV is an arbovirus, transmitted by mosquitoes, but unlike other known members of the *Flaviviridae* family, can also be transmitted sexually [29–31]. Although sexual transmission is not the main mode of ZIKV transmission, patients may be infected this way in non-endemic areas. Long term ZIKV persistence in semen after disappearance of disease symptoms increases the chances of sexual transmission. *In vivo* studies demonstrate that ZIKV can replicate in murine [17, 32, 33], hamster [34] and macaque testes [35, 36]. The virus caused severe inflammation and tissue destruction in murine testes [17, 32, 33], but similar effects have not been observed in humans. ZIKV can replicate in HSerC, which likely support viral persistence [18, 37], but the mechanism underlying this is not well understood.

We explored the impact of ZIKV infection on the HSerC proteome to complement earlier ZIKV-induced transcriptomic studies [18]. Unlike murine Sertoli cells [38], HSerC support high levels of ZIKV replication without any notable CPE up to 9 dpi (Fig 1A), as observed in other studies [18, 37]. ZIKV induced more dysregulation of the cellular proteome over time. Most dysregulated proteins were downregulated at 3 dpi whereas more proteins were upregulated at 5 dpi. Notably, at least 4 of these proteins (CASP3, HTRA2, SERPINE1, and SNCA) are associated with cell death, which is predicted to be increased by 3 dpi but decreased by 5 dpi. Thus, initially during active virus replication, cells were pushed towards cell death but later recovered, while virus titers started dropping after 5 dpi (Fig 1B and 1D). IPA also predicted similar observations based on the whole dataset analysis (S2 Table–S4 Table). These data are suggestive of a switch from an acute infection to a non-CPE persistent infection.

### ZIKV disrupts proteins involved in HSerC functions related to spermatogenesis

A large number of proteins known to play critical roles in HSerC functions, including innate immunity and spermatogenesis, were dysregulated by ZIKV infection. Pyruvate kinase (PKM2), an important regulator of the glycolysis pathway, was elevated in ZIKV-infected HSerC at 3dpi. Phosphoglycerate mutase 1 (PGAM1), another important regulator of glycolysis, was the most dysregulated protein at 5dpi. PGAM1 is a critical enzyme that plays important roles in cell proliferation, migration, and apoptosis [39]. Elevated expression often is associated with cancer [40–42] and spermatogenic dysfunction [39, 43].

Metalloproteinase inhibitor 1 (TIMP1) is a glycoprotein inhibitor of Matrix Metalloproteinases (MMPs) [44] that was expressed >10-fold higher in ZIKV-infected HSerC compared to mock. MMPs and TIMPs participate in matrix remodeling, fibrosis, semen liquefaction [44–47] and regulate Sertoli cell-tight junction dynamics [48]. TIMP1 is an important regulator for both testicular development and spermatogenesis [49]. TIMP1 was highly expressed in ZIKV-infected endothelial cells [50], is intimately involved in maintaining fetal membrane integrity until labor [51], and amniotic fluid levels decrease during pregnancy with advancing gestation [52]. Coordinated regulation of MMPs and TIMPs are required to maintain tissue architecture and normal ovarian function [53, 54]. Thus, ZIKV-induced abnormal expression of TIMP1 may hinder spermatogenesis, the ovulation process, and may cause premature fetal delivery. Thus, the impact of ZIKV infection on ovarian development and premature fetal delivery also needs to be investigated. However, the impact of over expression of TIMP1 on the BTB is not well understood as it regulates collagen degradation by a negative feedback mechanism [48].

Vitamin K-dependent protein S (PROS1), Stanniocalcin-1 (STC1), and Alpha-soluble NSF attachment protein (NAPA) were also among the top 5 most dysregulated proteins in ZIKV-infected HSerC. PROS1 is an anticoagulant cofactor to activated protein C in blood coagulation regulation [55], involved in leukocyte migration, apoptosis, and activation of complement [56]. Over expression of PROS1 may cause inhibition of macrophage-mediated killing of ZIKV-infected cells and protect the cells from apoptotic death. Stanniocalcin-1 (STC1) is an estrogen-regulated gene expressed by Sertoli, Leydig and spermatogonias in rat testes [55, 57] and important for testis development [58, 59]. STC1 also is strongly linked with pregnancy complications [60]. NAPA is critical for the fertility of both male and female mice [61, 62]. Further study is necessary to better understand the roles of STC1 and NAPA in ZIKV pathogenesis.

The IL-6 signaling pathway was predicted as the most significantly upregulated at 5dpi. Overexpression of IL-6 adversely affects differentiation during spermatogenesis [63] and stimulates disruption of the BTB [64]. IGF1 receptors play crucial roles in maintenance of testis size and sperm production [65]. *In vivo* studies showed ZIKV reduces murine sperm count and testis size [17, 32, 33]. Human sperm counts were reduced after ZIKV infection [66] but the impact on testis size is unknown. The IGF-1 signaling pathway was predicted to be highly activated in HSerC by 5dpi. Granulocyte macrophage-colony stimulating factor (GM-CSF), a growth factor that stimulates porcine spermatogonia survival [67], was also highly activated by 5dpi. Blocking epidermal growth factor (EGF) receptors suppresses spermatogonial proliferation in newts [68].

Apoptotic cell death induced by controlled activation of nuclear factor-κB (NF-κB) signaling is needed to balance testicular germ cell production in healthy individuals [69]. NF-κB expression is also dependent on spermatogenesis stages [70]. Overactivation of NF-κB signaling induces excess sperm killing and causes infertility [71]. This pathway was highly activated at 5dpi and could explain low sperm counts in ZIKV-infected semen. Fibroblast growth factor (FGF) signaling, which was also highly activated by ZIKV infection, is an important pathway for regulating the male reproductive system [72] and maintenance of undifferentiated spermatogonia [73]. Fibroblast growth factors are hormones that stimulate DNA synthesis and proliferation of Sertoli cells [74], and overexpression can cause testicular tumors [75]. A transcriptomic study also confirmed that fibroblast growth factor-2 (FGF2) gene was highly expressed in ZIKV-infected Sertoli cells and might be associated with viral persistence [18]. ZIKV infection has not been found associated with testicular tumors in any study to date, but the long-term effects are unknown, which needs further investigation. Higher activation of mitogen-activated protein kinases (MAPKs) might also adversely impact the function of spermatozoa and spermatogenesis as regulated by the pathway [76].

Germ cell energy homeostasis is regulated through Sertoli cell function [77]. The PPAR pathway plays an important role in Sertoli energy generation. For example, activation of the pathway is required for lipid droplet formation, lactate production and metabolism [77, 78]. Downregulation of the PPAR pathway might dysregulate Sertoli energy supplies and adversely affect spermatogenesis. IPA also predicted downregulation of lactate-dehydrogenase (LDH) levels and quantity of metal ions (Fig 4C). Lactate and pyruvate are critical substrates for energy generation supplied by Sertoli cells to spermatocytes [79, 80].

MicroRNAs (miRs) also play critical roles in male fertility and spermatogenesis by controlling germ cell gene expression [81–83]. Self-renewal of murine germ cells is regulated by mir-21 [84]. The absence of *miR-17-92* cluster genes in Sertoli cells can change the testicular phenotype with an alteration in the whole transcriptome [85]. Upstream IPA analyses predicted *miR-17* and *mir-*21 were significantly downregulated in Sertoli cells by ZIKV infection, which might have an adverse impact on spermatogenesis and testes morphology.

### ZIKV disrupts HSerC proteins involved in BTB tight junctions

Overactivation of the ERK/MAPK pathway disrupts the BTB, Sertoli–germ cell anchoring junction, and increases tight junction permeability [54, 86–88]. ERK pathway activation was also required for YFV replication [89]. ZIKV infection dysregulated the MAPK pathway in human neural progenitor cells [90] and in Sertoli cells by transcriptomic analysis [91]. The over activation of ERK/MAPK pathway indicates the possibility of BTB damage and disruption of this immune-privileged environment for the spermatogenesis process. The pathway might also be required for ZIKV replication, as observed in other members of the *Flaviviridae* [89, 92, 93]. Sertoli cell tight junctions are regulated by nitric oxide synthesis [94], and down regulation might impact BTB integrity. IPA also predicted nitric oxide synthesis was reduced by ZIKV infection through PPAR signaling.

Cytokine signaling can stimulate the breakdown of tight junctions by hindering junction protein synthesis [95]. IPA also predicted many cytokines, including IL-15, IL-27, IL-32, C5, IL-18, and EB13, were significantly up-regulated by ZIKV infection in HSerC. IL-15 was found elevated in the serum of ZIKV-infected patients [96], and important for maintenance of cellular tight junctions [97]. IL-18 and IL-23 levels in serum of ZIKV infected monkeys were also increased [98]. Testicular cells are specialized to control reactive oxygen species levels [99], but higher levels of hydrogen peroxide were predicted to accumulate in ZIKV-infected HSerC, which might be the cause of impaired regulation system. However, the ECIS results (Fig 2) demonstrate that CPE, and hence the predicted breakdown of HSerC tight junctions, takes considerably longer in HSerC than does CPE in permissive Vero cells, the model cells used for ZIKV propagation and titration, which could explain the long-lived persistence of ZIKV in testicular tissues and semen.

Sertoli cells secrete inhibin B, which regulates the expression of FSH, one of the critical regulator hormones for spermatogenesis. Pierik et al. showed that levels of serum inhibin B directly correlate with sperm count, which can be used as a marker for spermatogenesis. Low levels of inhibin B were detected in the serum of infertile males compared to healthy individuals [27]. ZIKV-induced reduction of inhibin B (Fig 5) might be the cause of dysregulated spermatogenesis, resulting in a decrease in sperm count, as observed in previous studies [66].

**Fig 6:**
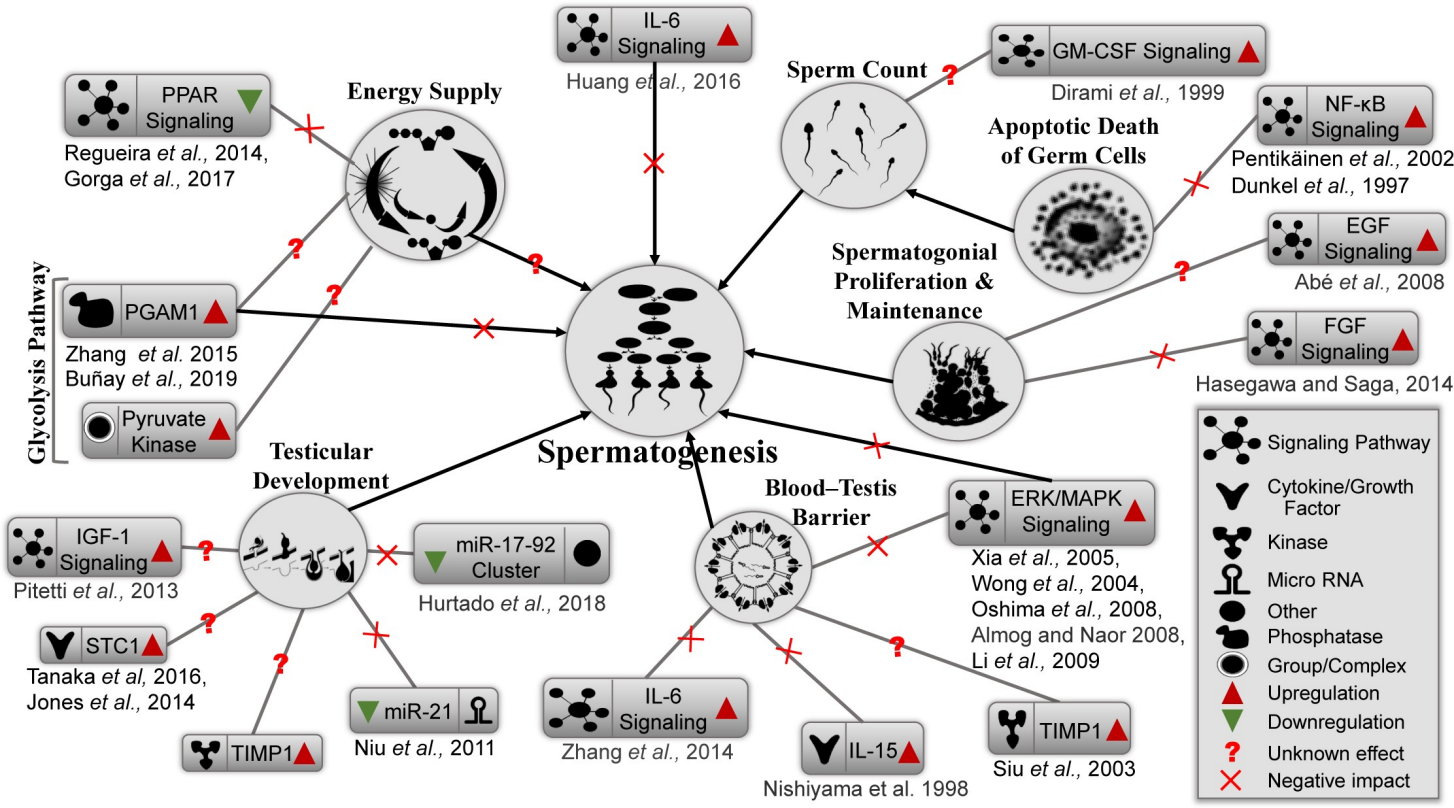
Proposed model for ZIKV-induced impairment of spermatogenesis. ZIKV-induced dysregulation of different proteins and cellular signaling pathways important for spermatogenesis. Energy supply is predicted to be affected by dysregulation of the PPAR signaling and glycolysis pathways. Testicular development could be affected by downregulation of microRNAs and upregulation of STC1, TIMP1, and IGF-1 signaling pathways. BTB integrity could be compromised by upregulation of cytokine (IL-6, IL-15 or ERK/MAPK) signaling pathways. Upregulation of IL-6 and ERK/MAPK signaling could also directly impact the spermatogenesis process. NK-kB and GM-CSF signaling could reduce the sperm count via apoptosis. However, spermatogonial proliferation and maintenance, regulated by EGF and FGF signaling pathway, were significantly upregulated by ZIKV infection, which could also adversely impact the spermatogenesis process.

In conclusion, Sertoli cells support every step of spermatogenesis by providing environmental support, supplying nutrients, and appropriate signals for proliferation and development of germ cells. Any adverse impact on Sertoli cells might affect overall spermatogenesis (modeled in Fig 6), which could result in male infertility. ZIKV infection of Sertoli cells can adversely impact different signaling pathways, bio-functions, cytokines, enzymes, and different cellular proteins that play important roles in spermatogenesis and BTB integrity. For example, activation of the PPAR pathway (Fig 6, upper left) is required for lipid droplet formation and lactate production and metabolism [77, 78, 100]. Thus, PPAR downregulation can negatively affect energy supply. In addition, PGAM1 (Fig 6, left) is a critical enzyme that regulates the glycolysis pathway and plays important roles in cell proliferation, migration and apoptosis [39]. Elevated expression is often associated with cancer [39] and spermatogenic dysfunction [39]. Further studies of these various affected pathways and processes are necessary for a deeper understanding of the impact of ZIKV infection on male and female fertility.

## Materials and methods

### Cells

Primary human Sertoli cells (HSerC**)** were purchased from ScienCell Research Laboratories, USA **(**Catalog #4520). Cells were grown in poly-L-lysine-coated culture vessels in Sertoli Cell Medium (ScienCell Cat. #4521) at 37°C in 5% CO_2_. Cells were passaged every 2–3 days by mild trypsinization as recommended by the company. Cells grown to passage 4 were used for all experiments.

### Virus

The Zika virus strain from Puerto Rico used in this study (ZIKV/Homo sapiens/PRI/PRVABC59/2015) was a gift from Dr. David Safronetz, Chief of Special Pathogens, the National Microbiology Laboratory, Public Health Agency of Canada. The virus stock was amplified by growing in Vero cells (ATCC #CCL-81) and preserved in 10% FBS at -80°C for future use. Stock virus titers were determined by plaque assay in Vero cells.

### Infection

HSerC were grown to 70% confluency and infected at a multiplicity of infection (MOI) of 0.3 or 3 plaque forming units (PFU) per cell, as detailed in specific experiments. The virus inoculum was adsorbed to cell surface by rocking the culture plates every 15 minutes for 2 hours at 37°C in a CO_2_ incubator, followed by adding fresh Sertoli cell medium. For viral growth curve determinations, supernatants of ZIKV-infected cells were collected every alternating day from day 1 to 9 post-infection. Virus titers in the supernatants were determined by plaque assay. All experiments were done in triplicates.

### Cell viability

Cell viability was determined using cell proliferation reagent WST-1 (Roche) according to the manufacturer’s protocol. Briefly, HSerC cells were grown in 96 well plates and infected with ZIKV at MOI = 3. Nine μl of WST-1 reagent was added to each well various days post infection and incubated at 37°C for 2 hours. Colorimetric changes were determined by photo-densitometer and cell viability was calculated compared to time-matched non-infected wells. Each experiment was done in 3 biological replicates with 5 technical replicates each time.

### Protein extraction and quantification

Assessment of proteomic dysregulation was performed by Western blot and SOMAscan-determined on ZIKV-infected and time-matched mock-treated cells on Day 1, 3 and 5 post-infection (dpi). Cells were scraped from the culture vessels, washed 3 times in ice-cold PBS and lysed by sonication in 60 μl MPER detergent, supplemented with 1× HALT Protease inhibitor solution. Insoluble cellular components were removed by centrifugation at 14,000×*g* for 10 min at 4°C. The concentrations of proteins in the clear supernatants were determined by BCA Protein Assay (Pierce; Rockford, IL) and quantified using bovine serum albumin standards.

### Immunoblotting

Protein concentrations from the cell lysates were measured as described above by BCA assay and 20μg of protein were resolved in SDS-PAGE gels and transferred to 0.2μm nitrocellulose membranes. 12% and 6% gels were used for separation of lower and higher molecular weight proteins, respectively. Rabbit anti-PSMA2 (Cell Signaling, Cat. 2455), anti-THBS2 (Abcam, Cat. ab84469), and anti–CST3 (Abcam, Cat. Ab109508), and mouse anti-ZIKV NS1 (BioFront Technologies Cat. BF-1225-06), anti-caspase 3 (Cell Signaling Cat. 3G2), anti-STAT1 (Cell Signaling, Cat. 9176S) and anti-Beta-Actin (Cell Signaling, Cat. 3700S) primary antibodies were used to detect specific proteins. (HRP)-conjugated horse anti-mouse (Cell Signaling, Cat. #7076) or anti-rabbit (Cell Signaling, Cat. #7074) secondary antibodies were used for detection of the immune complexes. Protein bands were imaged with an Alpha Innotech FluorChemQ MultiImage III after developed with ECL reagents. Band intensities were quantified with Image J 1.50i (USA) and graphically presented by Graphpad Prism software (La Jolla, California, USA).

### Photomicrography

ZIKV-infected and mock-treated HSerC cells were examined every alternate day from day 1 to 9 to observe cytopathic effects (CPE) of the virus infection with a Nikon TE-2000 inverted microscope and photographs were taken at 200× magnification with a Canon-A700 digital camera. Slight adjustments were made in brightness and contrast of the images in Power point, which did not alter image context with respect to each other.

### Immunofluorescent microscopy

HSerC cells were grown on spotted slides in Sertoli cell media at 37°C for 24 hours, and infected with ZIKV as previously described. Mock-infected cells were used as control. At 1, 3 and 5 dpi, both ZIKV-infected and mock cells were washed 5× with sterile 1× PBS and fixed with 4% paraformaldehyde for 15min. Paraformaldehyde was removed from cells by 5× wash with sterile 1× PBS. Cells were permeabilized with 0.1% TritonX-100 in PBS for 5 mins. 20 μl of 3% bovine serum albumin (BSA) was used overnight at 4°C for blocking. Cells were then incubated with primary anti-ZIKV-NS1 antibody in 3% BSA overnight at 4°C. After overnight incubation, cells were washed again 5× with sterile PBS and 0.2% Tween 20 (PBT) and incubated for 60min with Alexa Fluor546-tagged anti-mouse secondary antibody for 20min. Finally, cells were mounted with DAPI-containing mounting dye. Slides were imaged using a Zeiss Axio Observer Z1 inverted fluorescence microscope.

### SOMAscan analyses

A total of 18 (three biological replicates of each time point) cell lysate samples were collected from mock- and ZIKV-infected HSerC at 1, 3 and 5 dpi. For SOMAscan analysis, the protein concentrations were adjusted to 250 μg/ml. Proteomic analyses was performed on a SomaLogics-licensed platform in the Manitoba Centre for Proteomics and Systems Biology, using a SOMAscan version 1.3 platform capable of measuring 1307 proteins simultaneously from up to 92 samples. During the SOMAscan assay, each biologic sample was mixed with SomaLogic’s proprietary SOMAmers. Each of the SOMAmers has the capacity to selectively recognize and bind to a specific human protein [28, 101]. After mixing and binding each sample in an individual 96-well, the SOMAmers are washed, released, hybridized to DNA microarrays and quantified [101, 102]. The expression values are generated as relative fluorescent units (RFU) which are directly proportional to the amounts of target proteins in the initial samples, as confirmed by a standard curve generated for each protein-SOMAmer pair [28, 101].

### Electric cell-substrate impedance sensing of ZIKV infections

Cell barrier integrity was tested by electrical impedance as previously described by Nickol *et al*. [103]. Briefly, Vero and HSerC were added to 96-well ECIS plates (Applied Biosystems) and rested at 32°C/ 5% CO_2_ for 24 hours until confluent monolayers were formed. Cells were then treated with media alone (mock-infected positive control), 1% Triton X-100 (negative control), or with ZIKV at MOI of 0.3 or 3.0. Electrical impedance of the cultures was continually monitored from 24 hours before treatment to 90 hours post-treatment. Values for each treatment were determined from a minimum of 8 wells, and the HSerC were tested twice.

### Inhibin B levels detection

HSerC were infected with ZIKV (MOI 3) and induced with 62.5ng/ml or 125ng/ml follicular stimulating hormone (FSH) (Sigma-Aldrich, Canada, Cat. F4021). Mock-infected and FSH-treated or untreated HSerC were used as control. Cell culture supernatants were collected after 48 hours post-infection and stored at -20°C before analyzing. The inhibin B level was determined using manufacturers' recommendations, inhibin-B ELISA kit (Sigma-Aldrich, Canada, Cat. RAB0325). This assay has an inter-plate CV <15%, and intra-plate CV <10%, with minimum detection limit of 2pg/ml (n = 3 assays). The fold difference of inhibin B expression was determined by dividing by Mock-infected inhibin B concentration.

### Statistical and bioinformatics analyses

Data from the 18 ZIKV-infected and time-matched mock samples were imported into Excel and converted to Log_2_ values. Fold-changes of each of the proteins in each infected sample were determined by comparing each to its time-matched mock sample. Students T-test (2 tails) and Z-score analyses were done to quantify the p-value from the fold-changes, as described [104]. Average 1.3-fold and 1.5-fold dysregulation with p-value <0.05 were examined and cutoff of 1.3-fold was set for further analyses (S1 Table) by Ingenuity Pathway Analysis (IPA) and PANTHER. Any protein with a fold-change value beyond the 1.3-fold cutoff, but considered non-significant by T-test, was re-examined by Z-score analysis. The Z-scores from each replicate were determined, and cutoffs of ≥ 1.96σ or ≤ -1.96σ were considered significant. At least two of the replicates had to have significant Z-score values of the same trend (*e*.*g*. if up-regulated, two or more values must be >1.96σ, the 3^rd^ replicate must have a Z-score > 0.98σ, and the overall average must be ≥ 1.96σ). P-values for these were calculated from the average of the three Z-scores.

## Supporting information

S1 Fig Heat map of differentially expressed (fold change > 1.33 or < -1.33) proteins at 1, 3 and 5 days post ZIKV infection. Up- and down-regulated proteins are highlighted in red and blue, respectively.
(PDF)

S2 Fig Expression of significantly dysregulated proteins in molecular pathways at 1, 3 and 5 dpi. The datasets containing protein IDs, fold changes, and P-values were imported into IPA, and interacting pathways assembled for differentially expressed proteins. Up- and down-regulated proteins are indicated in red and green, respectively; gray were identified in this study but not affected; colorless proteins interact with various proteins in the pathway but are not recognized by the SOMA panel.
(PDF)

S3 Fig IPA-generated molecular networks of dysregulated proteins at 3 and 5 dpi. The datasets containing protein IDs, fold changes, and P-values were imported into the IPA software, and interacting networks were assembled for differentially expressed proteins at 3 and 5 dpi. Up- and down-regulated proteins are indicated in red and green, respectively; gray proteins denote those that were identified in this study but not dysregulated; colorless proteins interact with various proteins in the pathway but were not recognized in our screening.
(PDF)

S4 Fig Gene ontology (GO) analysis of up- and down-regulated proteins. Up- and down-regulated proteins were analysed by PANTHER and GOTERM database separately, and their associations to biological processes, molecular functions, and cellular components determined. Up-regulated and down-regulated protein functions at 3 and 5 dpi are listed in a, b, c, and d respectively.
(PDF)

S5 Fig Cellular impact of the most highly dysregulated proteins (± Fold change > 5.0, p ≤ 0.05) in ZIKV-infected HSerC. The most affected cellular network predicted by IPA at 3 dpi, shown at (**A**) 3 dpi and at (**B**) 5 dpi. (**C**) Disease and functions predicted significantly dysregulated at 5 dpi by most dysregulated proteins. (**D**) List of most dysregulated proteins at 3 and 5 dpi.
(PDF)

S1 Table List of proteins dysregulated at least 1.3-fold and significant by T-Test (p-value < 0.05) or Z-score (≥ 1.96σ or ≤ -1.96σ). dpi = days post infection, red = significantly up-regulated; green = significantly down-regulated; purple = p value < 0.05. Table sorted first by significantly up-regulated proteins at day 1 post-infection, then by those significantly down-regulated at 1dpi; then by significantly up- and down-regulated at 3dpi; then by significantly up- and down-regulated at 5dpi.
(XLSX)

S2 Table List of the most highly dysregulated IPA-determined "Diseases & Functions" at 3dpi and at 5dpi, with individual proteins assigned to each function depicted in red if significantly up-regulated, or in green if significantly down-regulated. Table sorted by each day, then according to default IPA score setting.
(XLSX)

S3 Table List of IPA-predicted significantly activated or inhibited Sertoli cell "Diseases and Functions" affected by ZIKV infection. Only those with predicted significantly affected activation or inhibition are indicated, in red or green, respectively. Table sorted first by whether Disease or Function activation state is decreased (in green) or increased (in red), then by p-value.
(XLSX)

S4 Table List of IPA-determined "Disease or Cellular Functions" significantly dysregulated by ZIKV infection at 5 dpi. Only those with predicted significantly affected activation or inhibition are indicated, in red or green, respectively. Table sorted first by Category name, then by p-value, then by whether Disease or Function activation state is increased (in red) or decreased (in green).
(XLSX)
