# Supplementary material for: Zika virus dysregulates human Sertoli cell proteins involved in spermatogenesis with little effect on tight junctions
Source: PLoS Negl Trop Dis. 2020 Jun 8;14(6):e0008335. doi: 10.1371/journal.pntd.0008335 (PMC7279580; doi:10.1371/journal.pntd.0008335)
Supplement: S1 Fig — Up- and down-regulated proteins are highlighted in red and blue, respectively. (PDF) [file pntd.0008335.s001.pdf]

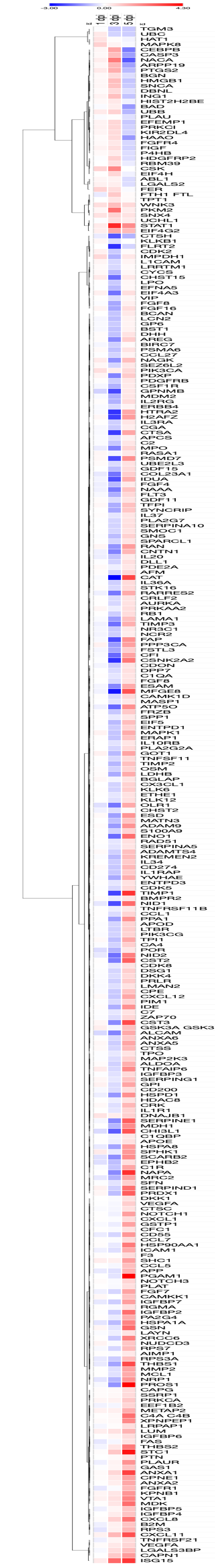

Supplementary Figure S1. Heat map of differentially expressed (fold change > 1.33 or < -1.33) proteins at 1, 3 and 5 days post ZIKV infection. Up- and down-regulated proteins are highlighted in red and blue, respectively.
