# Supplementary material for: Zika virus dysregulates human Sertoli cell proteins involved in spermatogenesis with little effect on tight junctions
Source: PLoS Negl Trop Dis. 2020 Jun 8;14(6):e0008335. doi: 10.1371/journal.pntd.0008335 (PMC7279580; doi:10.1371/journal.pntd.0008335)
Supplement: S2 Fig — The datasets containing protein IDs, fold changes, and P-values were imported into IPA, and interacting pathways assembled for differentially expressed proteins. Up- and down-regulated proteins are indicated in red and green, respectively; gray were identified in this study but not affected; colorless proteins interact with various proteins in the pathway but are not recognized by the SOMA panel. (PDF) [file pntd.0008335.s002.pdf]

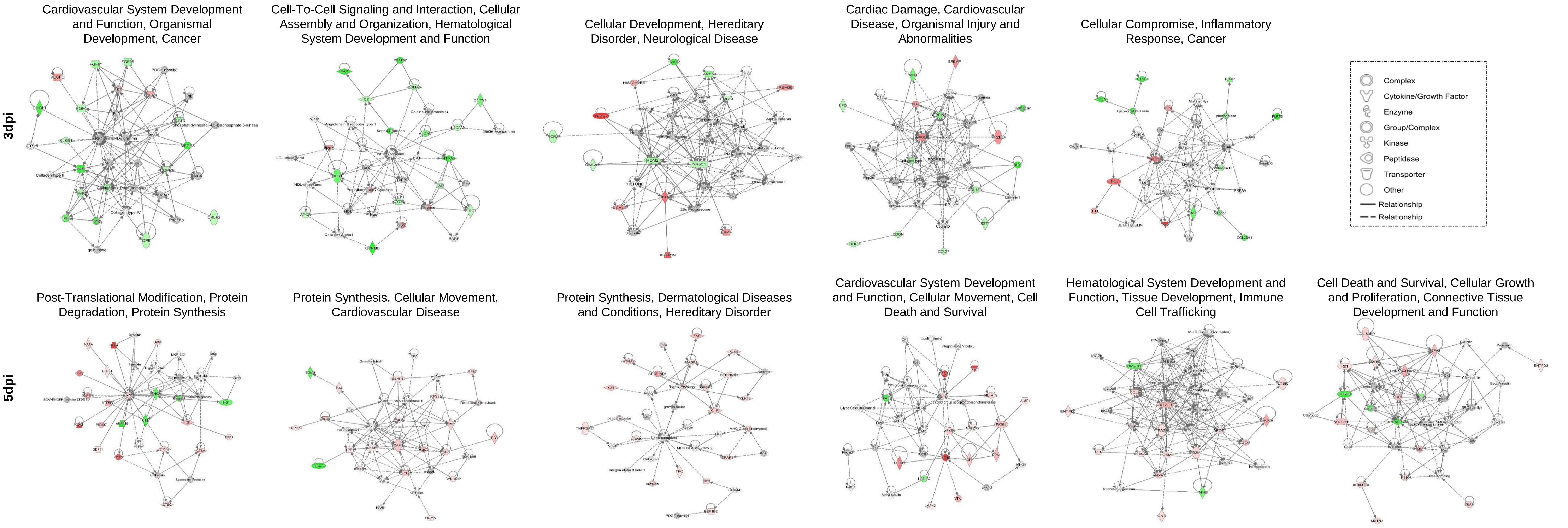

Supplementary Figure S2. IPA-generated molecular networks of dysregulated proteins at 3 and 5 dpi. The datasets containing protein IDs, fold changes, and P-values were imported into the IPA software, and interacting networks were assembled for differentially expressed proteins at 3 and 5 dpi. Up- and down-regulated proteins are indicated in red and green, respectively; gray proteins denote those that were identified in this study but not dysregulated; colorless proteins interact with various proteins in the pathway but were not recognized in our screening.
