# Supplementary material for: Zika virus dysregulates human Sertoli cell proteins involved in spermatogenesis with little effect on tight junctions
Source: PLoS Negl Trop Dis. 2020 Jun 8;14(6):e0008335. doi: 10.1371/journal.pntd.0008335 (PMC7279580; doi:10.1371/journal.pntd.0008335)
Supplement: S3 Fig — The datasets containing protein IDs, fold changes, and P-values were imported into the IPA software, and interacting networks were assembled for differentially expressed proteins at 3 and 5 dpi. Up- and down-regulated proteins are indicated in red and green, respectively; gray proteins denote those that were identified in this study but not dysregulated; colorless proteins interact with various proteins in the pathway but were not recognized in our screening. (PDF) [file pntd.0008335.s003.pdf]

Cell-To-Cell Signaling and Interaction, Cellular Assembly and Organization

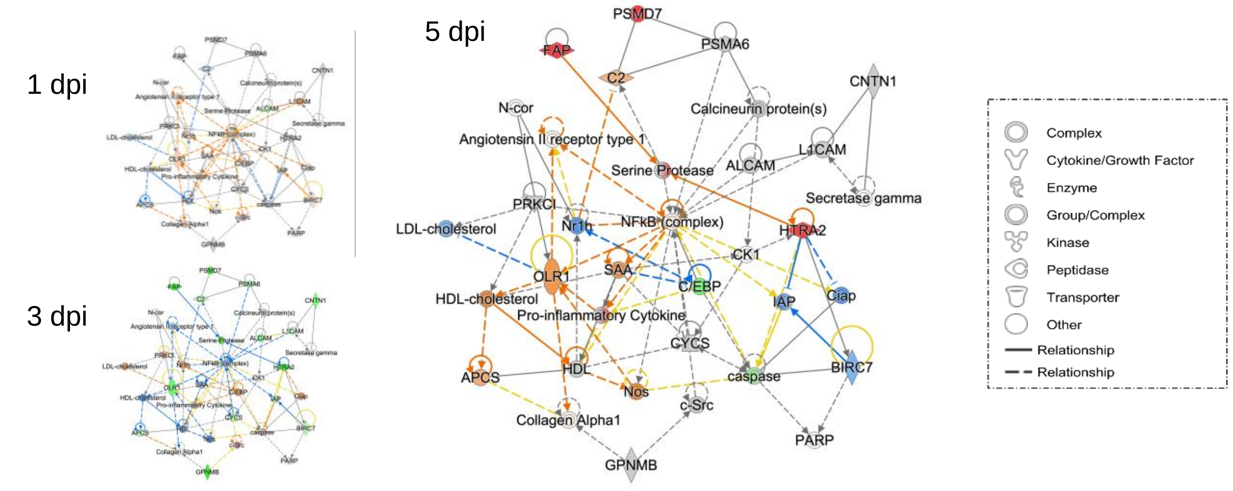

Cell Death & Survival, Cellular Growth & Proliferation, Connective Tissue Development & Function

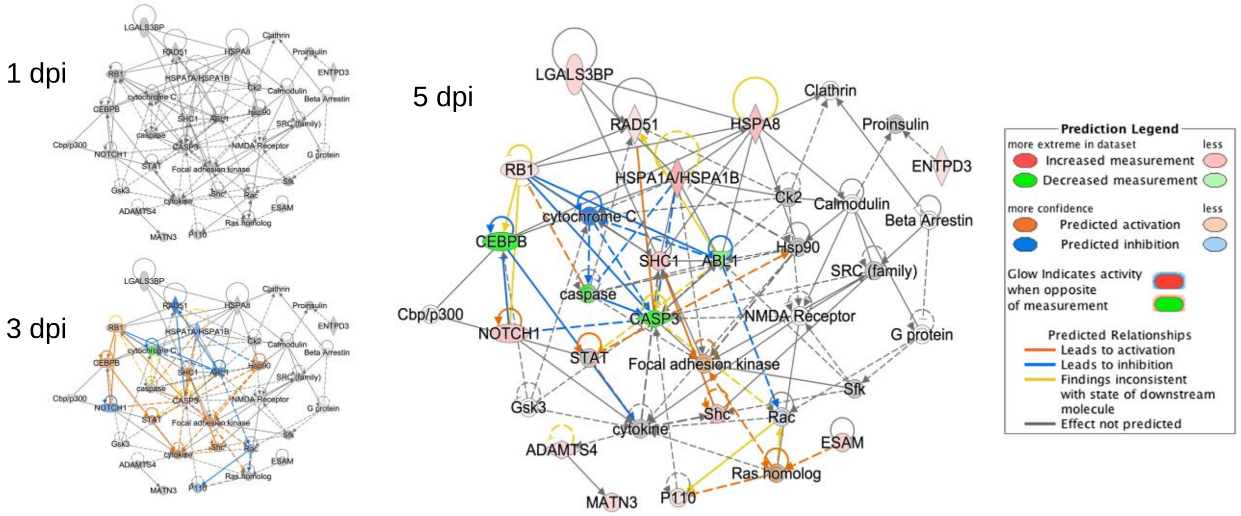

Post-Translational Modification, Protein Degradation, Protein Synthesis

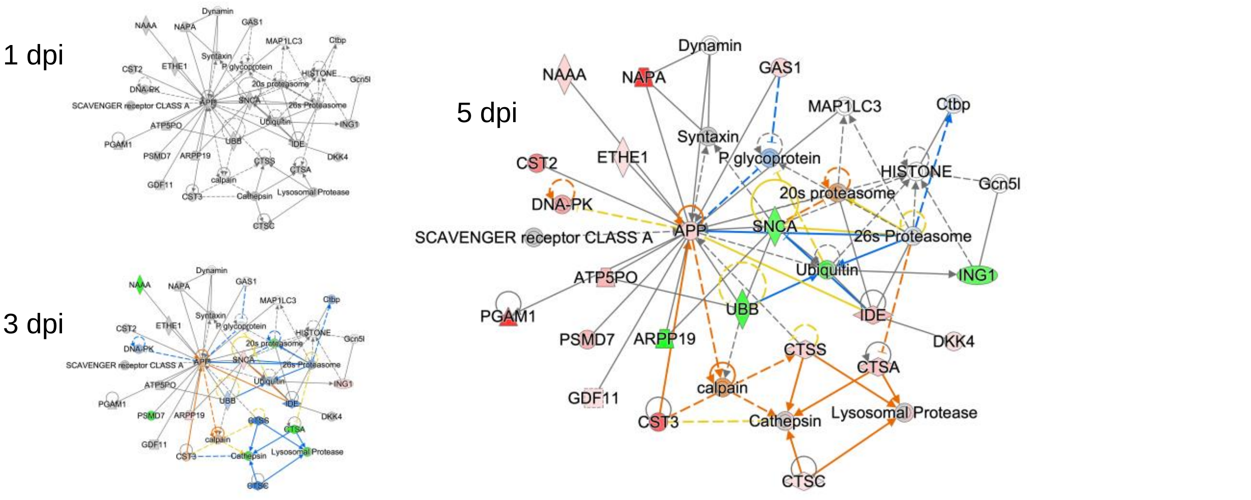

Supplementary Figure S3. Expression of significantly dysregulated proteins in molecular pathways at 1, 3 and 5 dpi. The datasets containing protein IDs, fold changes, and P-values were imported into IPA, and interacting pathways assembled for differentially expressed proteins. Up- and down-regulated proteins are indicated in red and green, respectively; gray were identified in this study but not affected; colorless proteins interact with various proteins in the pathway but are not recognized by the SOMA panel.
