# Supplementary material for: Zika virus dysregulates human Sertoli cell proteins involved in spermatogenesis with little effect on tight junctions
Source: PLoS Negl Trop Dis. 2020 Jun 8;14(6):e0008335. doi: 10.1371/journal.pntd.0008335 (PMC7279580; doi:10.1371/journal.pntd.0008335)
Supplement: S4 Fig — Up- and down-regulated proteins were analysed by PANTHER and GOTERM database separately, and their associations to biological processes, molecular functions, and cellular components determined. Up-regulated and down-regulated protein functions at 3 and 5 dpi are listed in a, b, c, and d respectively. (PDF) [file pntd.0008335.s004.pdf]

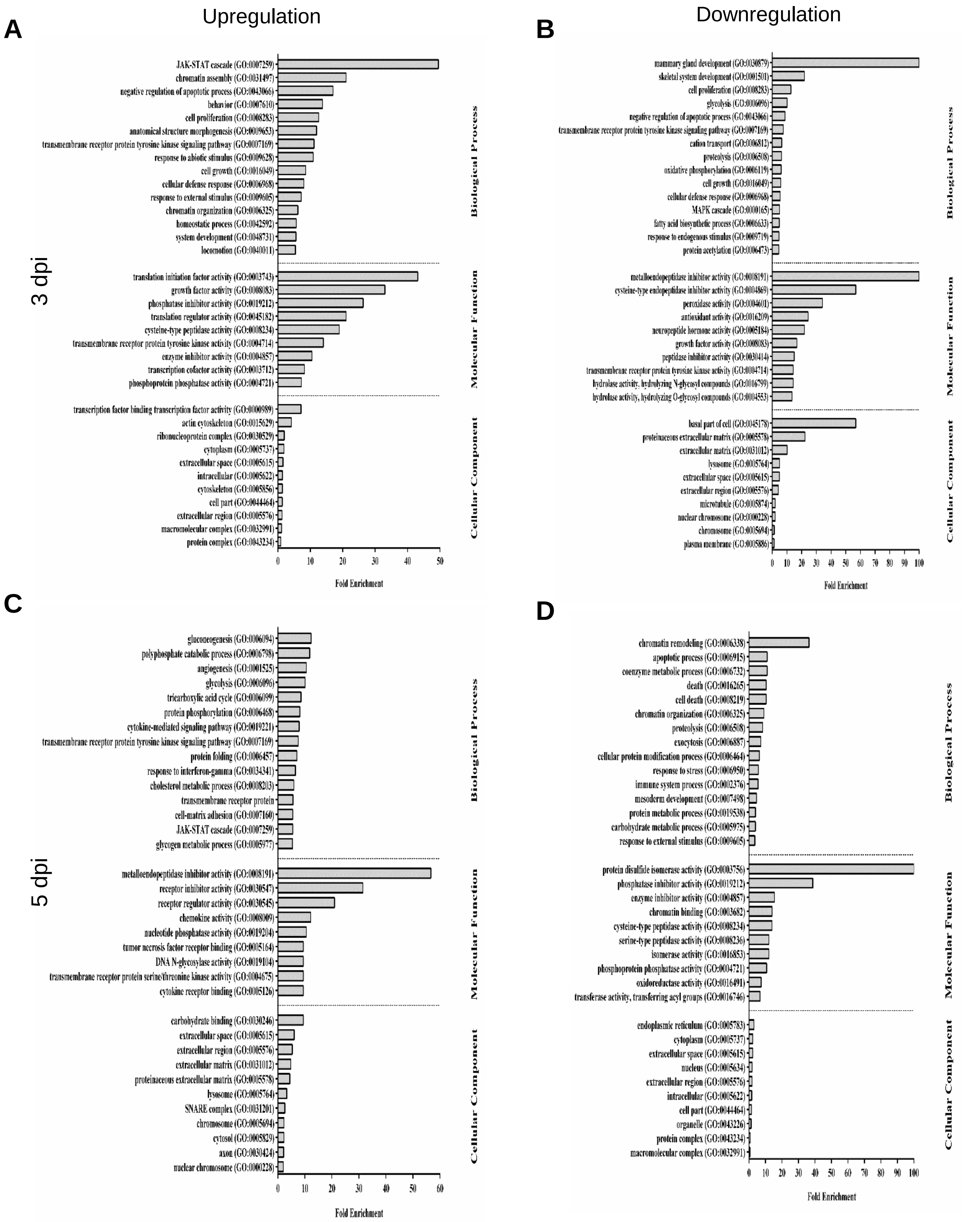

Supplementary Figure S4. Gene ontology (GO) analysis of up- and down-regulated proteins. Up- and down-regulated proteins were analysed by PANTHER and GOTERM database separately and their associations to biological processes, molecular functions, and cellular components determined. Upregulated and downregulated protein functions at 1, 3 and 5 dpi are listed in a, b, c and d respectively.
