# Supplementary material for: Zika virus dysregulates human Sertoli cell proteins involved in spermatogenesis with little effect on tight junctions
Source: PLoS Negl Trop Dis. 2020 Jun 8;14(6):e0008335. doi: 10.1371/journal.pntd.0008335 (PMC7279580; doi:10.1371/journal.pntd.0008335)
Supplement: S5 Fig — The most affected cellular network predicted by IPA at 3 dpi, shown at (A) 3 dpi and at (B) 5 dpi. (C) Disease and functions predicted significantly dysregulated at 5 dpi by most dysregulated proteins. (D) List of most dysregulated proteins at 3 and 5 dpi. (PDF) [file pntd.0008335.s005.pdf]

Cell-To-Cell Signaling & Interaction, Immunological Disease, Organismal Injury & Abnormalities

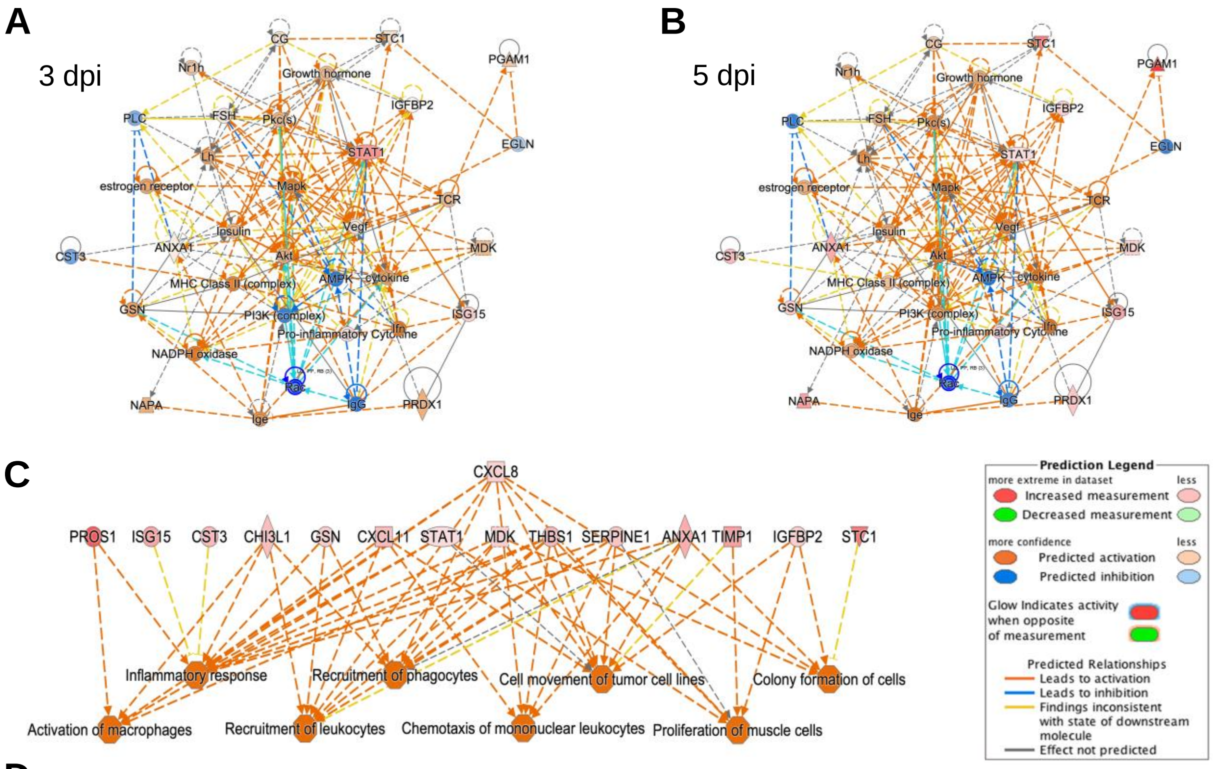

D

Disease and Functions (5dpi)

	Gene Symbol	Gene Name	Fold Change	P-value	Protein Type(s)
3 dpi	STAT1	signal transducer and activator of transcription 1	12.51	0.00002	transcription regulator
	ISG15	ISG15 ubiquitin-like modifier	5.76	0.0000014	other
	PKM	pyruvate kinase M1/2	5.41	0.0000032	kinase
	HTRA2	HtrA serine peptidase 2	-5.3	0.00014	peptidase
	FLRT2	fibronectin leucine rich transmembrane protein 2	-5.44	0.00011	other
	MFGE8	milk fat globule-EGF factor 8 protein	-6.75	0.00001	other
	CAT	catalase	-7.98	0.0000013	enzyme
5 dpi	PGAM1	phosphoglycerate mutase 1	23.24	0.038	phosphatase
	PROS1	protein S	18.92	0.0018	other
	STC1	stanniocalcin 1	15.41	0.000074	kinase
	NAPA	NSF attachment protein alpha	11.39	0.0012	transporter
	TIMP1	TIMP metallopeptidase inhibitor 1	11.02	0.044	cytokine
	ISG15	ISG15 ubiquitin-like modifier	10.4	0.0006	other
	ANXA1	annexin A1	9.23	0.0016	enzyme
	THBS1	thrombospondin 1	8.35	0.037	other
	CST3	cystatin C	8.16	0.012	other
	CXCL11	C-X-C motif chemokine ligand 11	7.73	0.00028	cytokine
	CHI3L1	chitinase 3 like 1	7.37	0.038	enzyme
	PRDX1	peroxiredoxin 1	6.66	0.025	enzyme
	SERPINE1	serpin family E member 1	6.56	0.014	other
	CST2	cystatin SA	6.5	0.014	other
	NID1	nidogen 1	5.74	0.015	other
	GSN	gelsolin	5.72	0.048	other
	MDK	midkine	5.45	0.00015	growth factor
	IGFBP2	insulin like growth factor binding protein 2	5.36	0.000071	other
	SERPIND1	serpin family D member 1	5.31	0.016	other
	CXCL8	C-X-C motif chemokine ligand 8	5.15	0.0028	cytokine

Supplementary Figure S5. Cellular impact of the most highly dysregulated proteins (± Fold change > 5.0, p ≤ 0.05) in ZIKV-infected Sertoli cells. The most affected cellular network predicted by IPA at 3 dpi, shown at (**A**) 3 dpi and at (**B**) 5 dpi. (**C**) Disease and functions predicted significantly dysregulated at 5 dpi by most dysregulated proteins. (**D**) List of most dysregulated proteins at 3 and 5 dpi.
